# Economics of One Health: Costs and benefits of integrated West Nile virus surveillance in Emilia-Romagna

**DOI:** 10.1371/journal.pone.0188156

**Published:** 2017-11-27

**Authors:** Giulia Paternoster, Sara Babo Martins, Andrea Mattivi, Roberto Cagarelli, Paola Angelini, Romeo Bellini, Annalisa Santi, Giorgio Galletti, Simonetta Pupella, Giuseppe Marano, Francesco Copello, Jonathan Rushton, Katharina D. C. Stärk, Marco Tamba

**Affiliations:** 1 Istituto Zooprofilattico Sperimentale della Lombardia e dell’Emilia-Romagna (IZSLER), Brescia, Italy; 2 Department of Production and Population Health, Royal Veterinary College, Hatfield, United Kingodm; 3 SAFOSO AG, Bern-Liebefeld, Switzerland; 4 Regional Health Authority of Emilia-Romagna, Bologna, Italy; 5 Centro Agricoltura Ambiente “G. Nicoli”, Crevalcore, Italy; 6 National Blood Centre, National Institute of Health (Istituto Superiore di Sanità, ISS), Rome, Italy; 7 Occupational Medicine Unit, IRCCS AOU San Martino-IST teaching Hospital, Genoa, Italy; 8 Institute of Infection and Global Health, University of Liverpool, Liverpool, United Kingodm; Uniformed Services University of the Health Sciences, UNITED STATES

## Abstract

Since 2013 in Emilia-Romagna, Italy, surveillance information generated in the public health and in the animal health sectors has been shared and used to guide public health interventions to mitigate the risk of West Nile virus (WNV) transmission via blood transfusion. The objective of the current study was to identify and estimate the costs and benefits associated with this One Health surveillance approach, and to compare it to an approach that does not integrate animal health information in blood donations safety policy (uni-sectoral scenario). Costs of human, animal, and entomological surveillance, sharing of information, and triggered interventions were estimated. Benefits were quantified as the averted costs of potential human cases of WNV neuroinvasive disease associated to infected blood transfusion. In the 2009–2015 period, the One Health approach was estimated to represent a cost saving of €160,921 compared to the uni-sectoral scenario. Blood donation screening was the main cost for both scenarios. The One Health approach further allowed savings of €1.21 million in terms of avoided tests on blood units. Benefits of the One Health approach due to short-term costs of hospitalization and compensation for transfusion-associated disease potentially avoided, were estimated to range from €0 to €2.98 million according to the probability of developing WNV neuroinvasive disease after receiving an infected blood transfusion.

## Introduction

In Emilia-Romagna region, Italy, West Nile virus (WNV) was detected for the first time in six horses with neurological symptoms in September 2008 in the vicinity of Ferrara province [1]. At the same time and in the same area, the first Italian human case of WNV neuroinvasive disease (WNND) was confirmed [2]. In total, three cases of WNND were confirmed in the region in 2008. Since 2009, an integrated and multi-disciplinary WNV surveillance system targeting humans, wild birds, horses, and mosquitoes has been implemented and adapted over time. The main objectives of this surveillance system are: (1) the early detection of WNV circulation in the environment; and (2) the mitigation of viral transmission risk via blood and solid organ donations [3].

From 2009 to 2012, the blood donations safety policy was based on a uni-sectoral approach. All blood donations in a given province were tested for WNV immediately after the first detection of a WNND case in that province until November 30, and also from July 1 to November 30 in the following year.

Since 2013, in agreement with the national blood authority, Emilia-Romagna has tentatively adopted a One Health approach to surveillance information use in blood donations safety policy [4–6]. Timely data on WNV detection generated by animal surveillance are integrated to trigger public health interventions on blood donors. Confirmation of WNV in any species targeted by the surveillance system in a given province activates the systematic individual Nucleic Acid Amplification (NAT) testing of blood donations in that province until the end of the annual transmission season (30 November), as requested by European regulation [7]. No additional testing is carried out during the following transmission season unless the surveillance system signals viral circulation again. This One Health approach is based on the cross-sectoral collaboration of different public institutions, resulting in a network linking human, animal, and environmental health, under the coordination of the regional working group on vector-borne diseases.

The implementation of surveillance systems and control measures for infectious diseases can be resource intensive activities. From an economic standpoint, the rational allocation of resources to those activities is based on the principle that associated overall costs are outweighed by benefits in terms of reduction of costs of diseases [8]. Economic assessment tools allow estimating the efficiency in terms of resource use of these approaches and guide policy and decision making of disease mitigation. The majority of this work focuses on uni-sectoral approaches to disease control and little has been carried out to establish the comparative efficiency in economic terms of One Health approaches to disease mitigation [8]. Yet, such information is key in the development of cost-effective zoonoses control, including for WNV.

The objective of the current study was to identify and estimate costs and benefits associated with the One Health approach used to guide blood donation safety policy for WNV in Emilia-Romagna in the 2009–2015 period.

## Materials and methods

**Fig 1:**
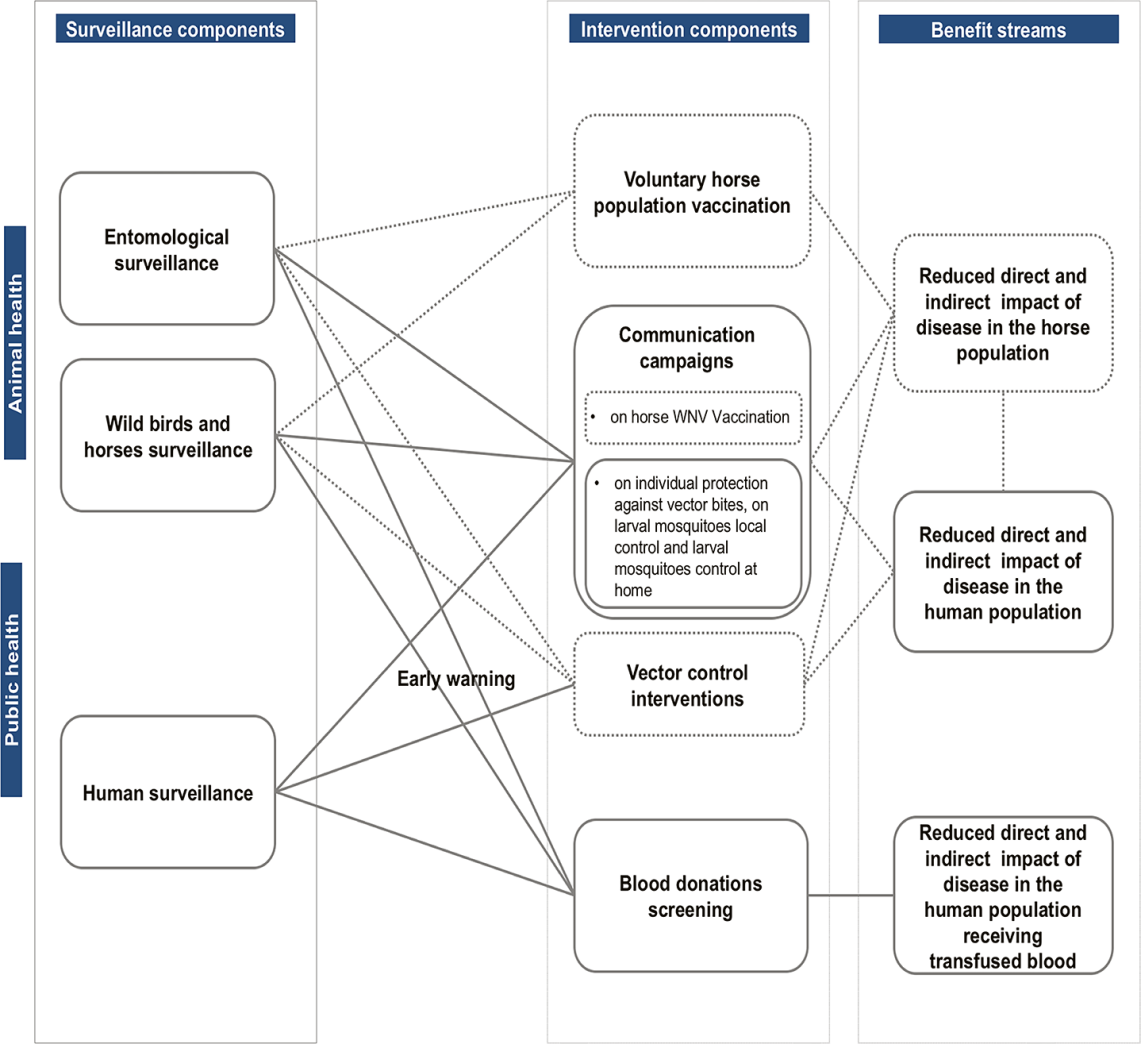
Identification of the links between West Nile virus (WNV) surveillance activities in the animal and human population, the wider public health disease mitigation system, and the benefit components associated, following the conceptual framework used in this analysis [9]. Dotted lines: elements identified but not considered in the economic evaluation. WNV integrated surveillance system in Emilia-Romagna, 2009–2015.

To guide the analysis a framework developed by Babo Martins *et al*. [9] was applied. The framework proposes the conceptualization of the links between surveillance activities across the two sectors and public health and animal health triggered interventions. Through such conceptualization, it was then possible to identify costs and benefit streams associated with surveillance activities (Fig 1). The next steps entail the valuation of the cross-sectoral costs and benefits identified. In this analysis, costs and benefits of surveillance designs and triggered interventions were estimated considering two scenarios. The One Health scenario currently implemented in Emilia-Romagna since 2013 as described in the introduction, and a uni-sectoral scenario, corresponding to the national regulation on blood safety. The latter triggers the activation of mandatory WNV testing with the confirmation of a human WNND or West Nile fever (WNF) case in a given province [10], and systematic testing is carried out in that province until the end of the vector season (30 November) and also between 1 July and 30 November in the following transmission season [11].

### Cost components and data sources

The Emilia-Romagna integrated WNV surveillance system includes human, entomological, ornithological, and horse surveillance, and triggered public health interventions (i.e. blood screening, vector control, and communication campaigns). Detailed description of this surveillance system has been previously provided by Angelini *et al*. [12] and Calzolari *et al*. [13]. Bellini *et al*. [3] performed a first cost evaluation of the regional integrated surveillance system for the 2009–2013 period. In this study, the same methodology was applied to update the costs of entomological and ornithological surveillance, and blood screening activity for 2014 and 2015, using the A and B scenarios identified in that work as the uni-sectoral and One Health scenarios, respectively. In addition to this, costs of human and horse surveillance, sharing of information, vector control interventions, and communication campaigns were estimated for the study period. For the One Health scenario, costs of human, entomological, ornithological, horse surveillance, and sharing of information were also considered. For the uni-sectoral scenario only the costs of human surveillance were included. For both scenarios, costs of the triggered public health interventions were estimated. The details for the estimation of these cost components are detailed below. Table 1 provides summarized information on all cost items considered in the analysis.

**Table 1:** Cost items included for the estimation of costs of the West Nile virus (WNV) integrated surveillance system in Emilia-Romagna, 2019–2015.

Item	Description	Value	Unit	Details	Source
**Surveillance activities**					
**Human surveillance**^a^	Mean cost of diagnosis of WNND on suspect cases: PCR, IFA, microneutralisation on blood and cerebrospinal fluid	74	Euro/case	Personnel not included, value added tax (VAT) included	[3]
**Horse surveillance**^**b**^	Mean cost of diagnosis of WNV neurologic disease on suspect horses: **Screening**: IgM ELISA on serum and PCR on EDTA-blood of alive suspect horses	20	Euro/case	Personnel and VAT included	[14]
	**Confirmation** of each positive sample: PCR, cell culture and sequencing	30	Euro/case		
**Ornithological surveillance**	Wild birds collection	50	Euro/consignment	Lump sum per consignment	[3]
	**Screening**: PCR on pool of organs (brain spleen heart and kidney) of individual wild birds	15	Euro/bird	Personnel and VAT included	[3] for 2009–2013; [14] for 2014–2015
	**Confirmation** of each positive pool: PCR, cell culture and sequencing	30	Euro/ pos. bird		
**Entomological surveillance**	**Screening**: PCR on each mosquito pool	15	Euro/pool		
	**Confirmation** of each positive pool: PCR, cell culture and sequencing	30	Euro/ pos. pool		
	Mosquito collection, species determination and pools preparation	Variable^c^	Euro/year	Lump sum for each season	[3] for 2009–2013; [15,16] for 2014–2015
**Sharing of information**	Mean number of meetings of the regional working group on vector-borne diseases per year	10	Number		Marco Tamba, personal communication March 2016
	Mean number of participants per meeting	10	Number		
	Mean duration of each meeting	4	Hours		
	Hourly wage of official veterinarians	56	Euro /hour		National Job Agreement for Public Health Sector wage
**Triggered interventions**					
**Blood testing**	Nucleic Acid Amplification Test (NAT) on each blood-donor sample	Variable^d^	Euro/test	Personnel not included, VAT included	[3]
**Communication campaigns**	Production and dissemination of informative brochures; design, update and maintenance of a website	15,000	Euro/year	Yearly funding for public information activities	[15,16]
**Vector control**	Average annual cost attributable to vector control interventions (ground adult mosquito control) triggered by the notification of human WNND cases	102,870	Euro/year	Average annual cost attributable to vector control interventions due to WNND in 2009	[17]

WNND: West Nile virus neuroinvasive disease; VBD: vector-borne diseases

^a^ The human surveillance system consists in case reporting and epidemiological investigation throughout the whole year overall regional territory, and in active identification of WNND human cases. According to the national case definition, every patient presenting fever (≥ 38.5°C) and a neurologic manifestation (i.e. encephalitis, aseptic meningitis or acute polyradiculoneuritis Guillain–Barre syndrome, or acute flaccid paralysis) is considered as a suspect, and is promptly reported to the Public Health Department to be tested for WNV [10].

^b^ Continuous syndromic surveillance for WNV neurologic disease in the horse population is carried out year-round overall the regional territory. Every horse presenting neurological signs (i.e. weakness, usually in the hind limbs, paralysis or partial paralysis, irregular gait, muscle twitching, proprioceptive deficits, impaired vision, flaccid paralysis of the lower lip, paralysis or partial paralysis of facial and labial muscles, teeth grinding) is considered as a suspect, it is mandatory reported to the veterinary authorities, and is tested for WNV [18].

^c^ The annual sum allocated for mosquito collection, species determination and pools preparation was €75,000 in the 2014–2015 period, €60,000 in 2013, and €50,000 for the 2009–2012 period.

^d^ The cost for a single NAT-test on a blood-donors sample was €11.32 in the 2013–2015 period, €12.10 in 2012, and €12.00 in the 2009–2011 period.

The cost of human surveillance was estimated for the study period considering the mean cost of diagnosis of WNND on a suspect case, times the number of suspect cases. Costs included value added tax (VAT), labour cost was not included [3]. The cost of horse surveillance was determined as the cost of diagnosis (screening and confirmation) of WNV in the study period, times the number of suspect cases. Costs included personnel and VAT, and were extracted from the invoice value of the official laboratory for WNV diagnosis in animals and vectors (IZSLER) price list [14].

Sharing of information to guide public health interventions to mitigate the risk of viral transmission via blood transfusion occur through regular meetings of the regional working group on vector-borne diseases, an epidemiologic bulletin periodically elaborated during the vector season, telephone, and email. The cost of information sharing was estimated considering the number and duration of the meetings, the number of participants, and the hourly wage of public health workers (i.e. official veterinarians). Costs associated with time used in communication and information sharing through bulletins, emails and phone calls could not be estimated.

Costs of communications campaigns were estimated as the yearly funding for public information activities provided through the regional deliberation on VBD throughout the study period [15, 16]. This funding was designated for the production and dissemination of informative brochures, and for the design, update, and maintenance of informative websites.

The difference was calculated between the mean expenditure allocated to ground adult mosquito control interventions after the notification of communicable diseases in Emilia-Romagna in a two-year period with no human WNND cases (2010–2011), and the same item of expenditure in a year characterized by the notification of human WNND cases (2009). It was assumed this difference could be attributable to vector control interventions triggered by the occurrence of human WNND cases. This expenditure was considered as the mean annual cost for vector control in those years characterized by the occurrence of WNND human cases. Data were obtained from the Emilia-Romagna report on the costs allocated in each local health unit (LHU) for vector control interventions within the regional arboviruses surveillance plan in the 2008–2011 period [17].

### Benefits considered and data sources

In the One Health scenario, surveillance data generated in the animal and vector population provided an early warning signal of WNV presence in these populations. In 2013 and 2014, this early warning enabled the detection of six infected blood donations in three provinces, before the onset of the first human cases of WNND or WNF in the same provinces. These infected blood donations would not have been tested for WNV following the national guidelines on blood safety (uni-sectoral scenario). Thus, marginal benefits of the One Health scenario were estimated as the averted costs of potential WNND cases associated to the transfusion of infected blood components intercepted in the One Health scenario only. Considering that most donations are whole blood units and each of these is systematically fractioned into three different blood components (red cells, buffy coat/platelets, and plasma), a number of 18 potential blood recipients could receive an infected transfusion. To the best of our knowledge, there is a lack of literature on the probability of developing neurologic disease after receiving a WNV infected transfusion. Thus, minimum, intermediate, and maximum values of 0.66%, 10%, and 100%, respectively, were assumed to consider three effect sizes or scenarios. In the best-case scenario, the probability of developing neurologic disease after infected transfusion was assumed to correspond to that after bites of infected mosquitoes (0.66%) [19]. An intermediate value of 10% was assumed considering that about 20% of infected persons develop fever and other clinical symptoms including headache, tiredness, and body aches [19]. Finally, in the worst-case scenario, a 100% probability of transfusion transmitted WNND was assumed considering that a high proportion of blood recipients are immunocompromised and many of them are subject to chronic or intensive transfusion treatments [10]. Therefore, benefits of the One Health scenario were calculated as the avoided short term cost of hospitalization and avoided compensation for transfusion-associated disease (TAD) of 0, 2, and 18 potential WNND cases, according to three effect sizes or scenarios for transfusion-associated disease mentioned above, respectively. Due to hospital data anonymization, it was not possible to collect further data on any additional costs related to WNND long term sequelae (e.g. home care, rehabilitation, durable medical equipment, medication or medical appointment) incurred by the WNND cases when discharged from the hospital. Our study only included hospitalization costs of WNND cases for the estimation of benefits. Benefits were calculated as averted costs related to avoided short term cost of hospitalization and avoided compensation for TAD of potential human cases of WNND associated to infected blood component transfusions. Cost items and hospital data considered for the estimation of benefits are reported in Tables 2 and 3, respectively. Additional data on WNND cases hospitalization is provided in S1 Table of supplementary information. Data on WNND cases were acquired by the Emilia-Romagna Region Public Health Service from surveillance activity, and were treated and analysed anonymously. The details for the estimation of benefits are described below.

**Table 2:** Items included in the calculation of avoided short term cost of hospitalization and avoided compensation for transfusion-associated disease for the estimation of benefits, West Nile virus (WNV) integrated surveillance system in Emilia-Romagna, 2009–2015.

Item	Description	Value	Unit	Details	Source
**Mean short term cost of hospitalization of WNV neuroinvasive disease (WNND)**					
**Hospitalization data**	Date of admission and discharge to and from each hospital ward	Variable	Date		Hospital discharge form (HDF) database
	Hospital ward type	Description	NA	Intensive therapy, neurology, infectious and tropical diseases, haematology, neurology-rehabilitation etc.	
	Duration of hospitalization in each hospital ward	Table 3	Days		
	Primary and secondary diagnosis during hospitalization	Description	NA		
**Hospitalization cost**^**a**^	Daily cost of intensive therapy ward	1450	Euro/day	Direct costs: 1100 EUR/day Indirect costs: 350 EUR/day	Francesco Copello, personal communication, March 2016
	Daily cost of other hospital wards	450	Euro/day	Direct costs: 350 EUR/day Indirect costs: 100 EUR/day	
**Mean compensation for transfusion-associated disease**					
**Anamnestic data of WNND notified cases**	Sex and profession, thirty-day follow up status, local health unit (LHU) of notification	Description	NA		Surveillance form for infectious diseases (SMI) database
	Symptoms onset date, date of notification	Variable	Date		
	Age at symptoms onset date	Variable	Years		
**Compensation for TAD**	Compensation for TAD according to the subject’s income class	Variable^b^	Euro/year for 15 years		[20,21]

NA: not applicable; TAD: transfusion-associated disease

^a^ including direct costs (medical personnel, materials, health service) and indirect costs (hospital structure, direction, offices, light, phone, gas and cleaning).

^b^ €10,000/year for 15 years for a subject retired or belonging to the lowest income class.

**Table 3:** Duration (days) of hospitalization of 52 West Nile virus neuroinvasive disease cases occurred in Emilia-Romagna, 2009–2015.

Type of hospital ward	No. of WNND cases	Mean duration of hospitalization (days)	Range (days)
**Intensive care**	4	32.8	7–73
**Infectious and tropical diseases**	29	13.2	2–55
**Other hospital wards**	33	29.5	1–184
**Total**	**52**	**28.6**	**3–215**

WNND: West Nile virus neuroinvasive disease

#### Short term cost of hospitalization

During the study period in Emilia-Romagna, each confirmed WNND case was notified in the surveillance form for infectious diseases (SMI) database and was identified by an anonymous id-code. This code allowed to link the hospital discharge form (HDF) with the human surveillance information. For all WNND cases, data on sex and profession, notification date, symptoms onset date, age at symptoms onset date, thirty-day follow up status, and history of blood transfusion or organ transplant in the 28 days before the symptoms onset date were obtained from the SMI database. All WNND cases hospitalization data (date of admission and discharge to and from each hospital ward, ward type, primary and secondary diagnosis) were obtained from the HDF database. Hospitalization records preceding the symptoms onset date were excluded from the analysis. Only the first hospitalization occurring after the symptoms onset date was considered, and the possible consecutive hospitalization records resulting from the change of the hospital ward. All primary and secondary diagnoses were checked to confirm that WNND was the cause of hospital admission. The hospitalization length of stay of each WNND case was calculated as the sum of the duration of the selected hospitalization events.

Short term cost of hospitalization was estimated for each WNND case considering the hospitalization length of stay in each different hospital ward (days) times its relative daily cost. Daily cost included direct costs (medical personnel, materials, health service) and indirect costs (hospital structure, direction, offices, light, phone, gas, and cleaning). These costs were indexed as information was collected and analysed in a public hospital of Genova for the year 2015 (Francesco Copello, personal communication, March 2016). This hospital was comparable, in organization and structure, to the hospitals of Emilia-Romagna region in which all patients were hospitalized. Eventually, based on a total of 76 hospitalization records of 52 out of 53 confirmed WNND cases notified in Emilia-Romagna in the study period, a mean short term cost of hospitalization of a WNND case was calculated.

#### Compensation for transfusion-associated disease

The value of the compensation for transfusion-associated disease was obtained from the Italian regulation [20,21]. According to the average age and occupation at the symptoms onset date of all confirmed WNND notified cases in Emilia-Romagna in the study period, the mean compensation for transfusion-associated disease for the appropriate income class was considered.

## Results

The estimates of the annual costs associated with WNV surveillance in Emilia-Romagna in the study period for the One Health and for the uni-sectoral scenarios are reported in Tables 4 and 5, respectively. Entomological and ornithological surveillance represent the greater annual surveillance cost. When implemented, blood screening and vector control represent the most expensive public health interventions of this disease mitigation system. The costs of communication campaigns and vector control interventions are the same in both scenarios, as they are related to the incidence of human cases that occurred in 2009, and in 2013–2015.

**Table 4:** Cost evaluation for the One Health scenario—regional integrated West Nile virus (WNV) surveillance system, Emilia-Romagna, Italy, 2009–2015.

Year	Cost of surveillance activities							Cost of triggered public health interventions					Overall surveillance cost (Euro)
	Human surveillance	Horse surveillance	Entomological surveillance		Ornithological surveillance		Sharing of information	Communication campaigns	Vector control	Blood screening			
	Cost of diagnosis of suspect WNND cases (Euro)	Cost of diagnosis of suspect WNV neurologic disease cases (Euro)	Mosquito collection cost (Euro)	Mosquito screening cost (Euro)	Bird collection cost (Euro)	Bird screening cost (Euro)	Meetings cost (Euro)	Communication cost (Euro)	Vector control intervention cost (Euro)	No. of blood units tested	No. of positive blood units detected	Blood screening cost (Euro)	
**2009** ^**a**^^**b**^	5772	1100	50,000	28,380	16,900	16,065	22,400	15,000	102,870	44,295	0	531,540	790,027
**2010** ^**a**^^**b**^	8362	240	50,000	34,770	11,550	12,180	22,400	15,000	0	11,679	0	140,148	294,650
**2011** ^**a**^	4884	80	50,000	23,325	14,650	12,810	22,400	15,000	0	0	0	0	143,149
**2012** ^**a**^	5476	220	50,000	28,815	15,500	18,480	22,400	15,000	0	0	0	0	155,891
**2013** ^**c**^	14,726	270	60,000	39,510	18,400	29,880	22,400	15,000	102,870	74,242	12	840,419	1,143,475
**2014** ^**c**^	16,798	230	75,000	49,455	15,600	24,990	22,400	15,000	102,870	83,794	2	948,548	1,270,891
**2015** ^**c**^	15,170	200	75,000	32,250	15,800	22,515	22,400	15,000	102,870	72,058	6	815,697	1,116,902
**Total**	**71,188**	**2340**	**410,000**	**236,505**	**108,400**	**136,920**	**156,800**	**105,000**	**411,480**	**286,068**	**20**	**3,276,352**	**4,914,985**

WNV: West Nile virus; WNND: West Nile neurinvasive disease

Costs of entomological and ornithological surveillance, and blood screening activities for the years 2009–2013 are from Table 5 of Bellini *et al*. [3].

Blood screening activities

The integrated WNV surveillance system has been implemented during the whole study period in the Emilia-Romagna region. However, only the results of human surveillance were taken into account to trigger blood screening activities until 2013, following the national regulation (uni-sectoral scenario). In 2013, according to the regional surveillance system, WNV nucleic acid testing (NAT) screening is applied to all blood donors in a province after reports of at least two positive mosquito pools or one positive bird by the entomological or ornithological surveillance, within the limits of that province [3]. In 2014 and 2015 NAT screening at the province level is started after the confirmation of WNV in any species targeted by the surveillance system in that province. Therefore, for this scenario, blood screening data are estimated for 2009–2012, and real data for 2013–2015, based on the actual number of blood units tested and detected as positive.

^a^ In this year, blood screening surveillance in Emilia-Romagna does not follow the regional integrated WNV SS, but the national WNV surveillance plan. However, based on surveillance results, it is possible to predict how many blood units would have been screened should the ER surveillance system (OH scenario) have been followed. Costs were derived accordingly.

^b^ In this year, the blood units that would have been screened by the integrated WNV regional surveillance system happened to have been screened according to the national surveillance plan, so the number of positive blood units that would have been detected via the integrated WNV regional surveillance system is known.

^C^ In this year, blood screening activities are based on the results of the integrated SS. Blood screening data are based on the actual number of blood units tested and detected as positive.

**Table 5:** Cost evaluation for the uni-sectoral scenario–national plan for West Nile virus (WNV) surveillance, Emilia-Romagna, Italy, 2009–2015.

Year	Cost of surveillance activities	Cost of triggered public health interventions					Overall surveillance cost (Euro)
	Human surveillance	Communication campaigns	Vector control	Blood screening			
	Cost of diagnosis of suspect WNND cases (Euro)	Communication cost (Euro)	Vector control intervention cost (Euro)	No. of blood units tested	No. of positive blood units detected	Blood screening cost (Euro)	
**2009**^**a**^	5772	15,000	102,870	35,552	0	426,624	550,266
**2010**^**a**^	8362	15,000	0	66,689	0	800,268	823,630
**2011**^**a**^	4884	15,000	0	60,258	0	723,096	742,980
**2012**^**a**^	5476	15,000	0	0	0	0	20,476
**2013**^**b**^	14,726	15,000	102,870	53,898	8	610,125	742,721
**2014**^**b**^	16,798	15,000	102,870	79,554	0^c^	900,551	1,035,219
**2015**^**b**^	15,170	15,000	102,870	90,775	6	1,027,573	1,160,613
**Total**	**71,188**	**105,000**	**411,480**	**386,726**	**14**	**4,488,238**	**5,075,906**

WNND: West Nile virus neuroinvasive disease

Costs of blood screening activities for the years 2009–2013 are from Table 4 of Bellini *et al*. [3].

Blood screening activities

According to the national guidelines, mandatory screening of blood units is performed in a given year between 1 July and 30 November in all blood donations of provinces (NUTS 3) where a human case of WNND has been confirmed in the previous year. For other provinces that year, blood unit screening is only initiated a week after a human WNND case is detected in the current season [3]. Moreover, nationwide, from 1 July to 30 November, blood donors having been for at least one night in the affected provinces shall be deferred for 28 days, alternatively, they can be admitted to donate provided their donations are tested by WNV NAT.

^a^ The national regulation on blood safety is implemented in Emilia-Romagna during the year in question. Only the results of human surveillance trigger public health interventions to mitigate the risk of WNV transmission via blood transfusion (uni-sectoral scenario).

^b^ From 2013, in ER, surveillance information generated in the animal health sector is integrated to guide public health interventions to mitigate the risk of WNV transmission via blood transfusion (One Health scenario), differing from the national guidelines (uni-sectoral scenario). Costs that would have been generated by following the national guidelines have been estimated for the region, based on the knowledge of the notification of human WNND cases in the previous year, and the knowledge of the time of occurrence of human WNND cases at the province (NUTS3) level.

^c^ In 2014 the National Blood Centre established that in Emilia-Romagna region, having an integrated surveillance system in place, blood donation testing is implemented in a province from July 1, if WNV circulation has been detected in any species in the same province in the previous two years. According to this, one infected blood donation would have been detected in the province of Ravenna (2014) following the national regulation. However, we considered 0 donations detected according to the uni-sectoral national regulation implemented until 2013.

Cumulative nominal costs of surveillance activities and triggered interventions in the 2009–2015 period are depicted in Table 6. The overall costs of the One Health and of the uni-sectoral scenario were between €4.91 and 5.08 million, respectively. Blood donation testing was the main cost component in in both scenarios, and was estimated to be €4.49 million in the uni-sectoral scenario, and €3.28 million in the One Health scenario. The One Health blood donation strategy resulted in €1.21 million savings in terms of avoided tests.

**Table 6:** Overall costs of the One Health and uni-sectoral scenarios, Emilia-Romagna, Italy, 2009–2015.

	One Health scenario cost (Euro)	Uni-sectoral scenario cost (Euro)
**Surveillance activities**		
Human surveillance	71,188	71,188
Entomological surveillance	646,505	0
Wild birds surveillance	245,320	0
Horse surveillance	2340	0
Sharing of information	156,800	0
**Triggered interventions**		
Blood testing	3,276,352	4,488,238
Communication campaigns	105,000	105,000
Vector control interventions	411,480	411,480
	**4,914,985**	**5,075,906**

A mean short term cost of hospitalization of €15,396 per WNND case was calculated, referring to a mean hospitalization length of 28.6 days. Compensation for transfusion-associated disease for a low income or retired subject, which was the case of 46/53 WNND cases (mean age at symptoms onset: 70.0 years), was estimated to be €150,000 in fifteen years per subject. In the study period, benefits due to avoided potential cases of WNND associated to infected blood component transfusion in the One Health scenario were estimated to be €0, €330,792, and €2.98 million in the best-case, intermediate, and worst-case scenario, respectively (Tables 7 and 8). These estimated benefits were due, in this analysis, to the detection of six infected blood donations (18 potential WNV infected transfusion and zero, two, or 18 associated WNND cases) that would have been undetected with the uni-sectoral approach.

**Table 7:** Benefits of the One Health scenario: Parameters included in the calculation of the averted costs of potential human cases of West Nile virus neuroinvasive disease (WNND) associated to infected blood component transfusion, Emilia-Romagna, Italy, 2009–2015.

Parameter description	Value	Unit
Number of infected blood units intercepted in the One Health scenario only	6	Number
Number of assumed WNND cases avoided in the One Health scenario only	Table 8	Number
Number of confirmed WNND cases notified in Emilia-Romagna in 2009–2015	53	Number
Number of confirmed WNND cases in Emilia-Romagna in the study period with hospitalization records	52	Number
Number of hospitalization records considered in the estimation	76	Number
Mean hospitalization length of a WNND case	28.6	Days
Mean short term cost of hospitalization of a WNND case	15,396	Euro
Mean compensation for transfusion-associated disease per subject	150,000^a^	Euro

WNND: West Nile virus neuroinvasive disease

^a^ Compensation in fifteen years.

**Table 8:** Benefits of the One Health scenario quantified as averted costs of potential human cases of West Nile virus neuroinvasive disease (WNND) associated to infected blood component transfusion. Best-case, intermediate, and worst-case scenario according to the probability of WNND transfusion associated transmission. Emilia-Romagna, Italy, 2009–2015.

	Best-case scenario	Intermediate scenario	Worst-case scenario
**Short term cost of hospitalization avoided (Euro)**	0	30,792	277,128
**Compensation for transfusion-associated disease avoided (Euro)**	0	300,000	2,700,000
**Total benefit of the One Health scenario (Euro)**	**0**	**330,792**	**2,977,128**

WNND: West Nile virus neuroinvasive disease

Benefits of the One Health scenario are estimated as potential transfusion associated West Nile virus neuroinvasive disease (WNND) cases avoided. Three scenarios are considered based on the assumed probability of developing WNND after receiving an infected blood transfusion. This probability was assumed to be 0%, 10%, and 100% in the best-case, intermediate, and worst-case scenario, resulting in 0, 2, and 18 potential WNND cases avoided, respectively.

## Discussion

Our estimates suggest that the One Health approach to WNV surveillance is cost saving. A total of €160,921 cost reduction can be achieved with the One Health scenario when compared to the uni-sectoral scenario considered in this work. Benefits of the One Health approach due to avoided short term costs of hospitalization and avoided compensation for transfusion-associated disease of potential WNND human cases were estimated to be €0, €330,792, and €2.98 million in the best-case, intermediate, and worst-case scenario, respectively.

Enhanced cooperation of human and animal health sector is considered to be crucial in order to improve the management of zoonoses, including WNV. Such cooperation enables the adoption of targeted measures and is expected to be more efficient and resource-saving, compared to a non integrated approach [22]. In a context of increasing pressure on public and private resources allocated to surveillance and intervention activities aimed at reducing the impact of zoonoses in society [9], it is worthwhile considering the economic implications of different public health strategies. In this work, the One Health approach was more efficient from an economic standpoint than the uni-sectoral approach. In fact, although the integrated WNV surveillance was resource intensive, the savings associated with the One Health approach compensated the costs of this surveillance system. These savings were due to a more targeted, evidence-based testing strategy, triggered by real-time area-specific WNV detection by the integrated surveillance system. Moreover, the One Health approach provided an early and timely warning of WNV presence in the animal and vector populations at the province level. This enabled the interception of infected asymptomatic blood donors (otherwise undetected) in the same space frame, resulting in substantial benefits associated to averted potential WNND cases due to infected blood component transfusion.

To our knowledge, studies of the comparative economic efficiency of One Health approaches compared to uni-sectoral approaches to vector-borne zoonoses management remain scarce and largely absent from the published literature. Previous work conducted to estimate the economic aspects of vector-borne zoonoses surveillance, prevention and control, have focused so far on uni-sectoral approaches, and conducted mainly in the United States (US). Few studies on the economic burden of WNV infection, including financial consequences of WNND and vector control interventions, have been carried out in Europe. In our study, a mean short-term cost of hospitalization of €15,396 per WNND case was estimated, with a mean hospital stay of 28.6 days. Humblet *et al*. [23] estimated the impact of a potential epidemic in Belgium, finding a cost of €3553 for a WNND case, and the €4441 for an acute flaccid paralysis case, considering a mean hospitalization length of nine days. Adjusting for hospitalization length, our results are similar to those obtained in that study. Kolimenakis *et al*. [24] performed a cost-benefit analysis of the public control strategies to tackle the 2010 WNV outbreak in the Region of Central Macedonia, Greece. The authors estimated an average cost of illness of about €4000 per hospitalised patient, including hospitalization costs and productivity losses. This estimation was lower compared to our findings, and was based on lower daily hospital care cost in Greek hospitals. Our estimates are in line with the US study of Staples *et al*. [25], that quantified a median cost (i.e. initial hospital and lost productivity costs) of US$20,774 (approximately €18,715, considering the European Central Bank yearly average exchange rate in 2015 of €1.11 to US$1 [26]) for a patient with neurologic disease. The majority of this cost was incurred as a result of hospital charges. Barber *et al*. [27] estimated a cost of US$33,143 (€28,094, value updated to the European Central Bank yearly average exchange rate in 2015 would be €29,859) including inpatient treatment costs but not including outpatient care, lost productivity, and miscellaneous expenses. A US$27,500 (€24,775) cost per case of neuroinvasive WNV illness with full recovery was estimated in the works on the 2002 WNV epidemic in Louisiana [28,29].

Concerning vector control intervention, a mean annual cost of ground adult mosquito control interventions of €102,870 was obtained. Our estimate is lower to the annual cost of about €4 million to tackle the 2010 WNV outbreak in Greece [24]. Similar studies would be needed to add this sort of information to the body of knowledge, as these are important aspects for post epidemic strategy decision making. Several works have been carried out in the USA. [27] estimated a total cost of US$701,790 (€632,243) for the mosquito control aerial spray after an human outbreak in the Sacramento County, California in 2005. The cost of mosquito control of the 2002 WNV epidemic in Louisiana was estimated to be US$8.3 million (€7.5 million) [29]. To our knowledge, these costs are not comparable, and aerial spray would unlikely be implemented in the European context, because of its heavy environmental impact [30].

There are several limitations to our estimations and areas where overestimation or underestimation of costs and benefits are possible, mainly due to a lack of data. For the hospitalized WNND cases, only a 30-day follow up after hospital discharge was available. Thus, long term cost of illness (e.g. home care, rehabilitation, durable medical equipment, medication, medical appointments) was not calculated at this stage. Furthermore, costs associated with the death of patients (compensation paid to the family, additional possible compensation requests within instances of transfusion associated diseases, private insurance claims) were not considered for a lack of data. Indirect impact of WNND (e.g. work absenteeism) was not quantified. However, it was assumed that this cost would not have a big impact on our benefit estimation, considering the mean age at symptoms onset of our cases (70.0 years). Costs were valued in nominal prices. There has been no attempt to adjust for inflation over the period because in both scenarios inflation rates would have been similar or the same, and the levels of inflation during the period were low. Averted compensation for potential transfusion-associated disease was the major benefit of the One Health scenario. Further benefits resulting from the averted costs related to legal actions after transfusion-associated disease were not quantified and included for a lack of data. Supplemental intangible costs attributable to the loss of reputation of the regional public health service, due to the occurrence of a human case after an infected transfusion, were not quantified at this stage. Similarly, any benefit streams associated with the reduced impact of disease in the horse population were not quantified.

Additional limitations are linked to the assumption that a WNV positive blood component donation always resulted in infection, as in the work of Korves *et al*. [31], and to the assumption of the probability of developing WNND after infected transfusion. A higher risk of disease in patients who were infected via blood transfusion based on their age and immune system status was not taken into account for a lack of these data. To the best of our knowledge, there is a lack of literature on the probability of disease transmissibility via blood transfusion. Thus, to address the possibility of different probabilities of TAD, three scenarios were considered using 0%, 10%, and 100% probability values. However, even considering a 0% probability of developing WNND in patients receiving a WNV positive blood component donation, the One Health scenario would still be economically beneficial, due to savings in surveillance activities (Table 6).

The estimation of blood screening cost might be an underestimation, as donor notification, retrieval of a test-positive sample, and personnel costs were not included as in the work of Korves *et al*. [31]. A cost for a single NAT-test on a blood-donor’s sample ranging from €11.32 (2013–2015), to €12.00 (2009–2011), and €12.10 (2012) was found. This estimation would be less than the €18 for single WNV-NAT test, including personnel and other overhead costs, estimated by Niederhauser *et al*. [32].

Human surveillance costs might have been underestimated as personnel cost was not included in the quantification of the mean cost of diagnosis of a human neurologic case (€74), due to a lack of data. The costs related to veterinary inspection and outbreak investigations after the confirmation of an equine WNV neurologic disease case was not quantified, because these activities did not have additional consequences on public health interventions. For the same reason, costs incurred by the horse owner (e.g. sample transportation to the laboratory, voluntary vaccination) were not included in this study.

Allocation of costs to specific WNV-related activities was not always possible. This resulted in a possible underestimation of information sharing cost. In fact, the time used to share surveillance data via email, telephone, and the epidemiologic bulletin could not be quantified. Communication campaigns cost might have been overestimated because communication materials were designed to inform the general population on the prevention of vector borne diseases (e.g. dengue, chikungunya, zika, leishmaniasis, etc.) in which WNND is included.

Although human infection is most often the result of bites from WNV infected mosquitoes, it is of utmost importance for public health decision makers to ensure the absence of viral transmission through hospital practices (i.e. blood transfusion and organ transplant) in order to maintain the trust in the public health system. During the 2009–2015 period, no WNND cases associated to a WNV infected blood transfusion were recorded in Emilia-Romagna. In fact, none of the hospitalized WNND received blood transfusions or organ transplants in the 28 days before symptoms onset. Breast feeding and laboratory related exposure were excluded considering the age and profession of all WNND cases. These data support the efficacy of the blood safety policy adopted in Emilia-Romagna region.

Better quality data, namely on long term costs of human disease, communication campaigns and vector control activities and voluntary horse vaccination is needed to more accurately assess the economic impact of WNV infection. Further economic assessments including long term costs of human disease, and the evaluation of intangible costs and sociological and ecological dimensions would allow a more complete understanding of the impact of the disease as well as of the economic efficiency of different disease mitigation strategies.

Blood screening was the main cost associated to WNV surveillance in this endemic region. It is therefore essential to invest in quantitative and qualitative research on the evaluation of different blood safety strategies and their economic impact. The One Health blood safety strategy is cost saving in our case. Moreover, it can enable an early warning of WNV presence in the environment, and the prevention of potential human infections via infected blood transfusion, resulting in substantial benefits.

Our results provide important evidence on the economic return of One Health surveillance for vector-borne zoonoses in Europe, and can further the evidence for the prioritization of resources allocated to zoonoses surveillance and prevention within a One Health context.

## Conclusion

Emilia-Romagna is a WNV endemic area. In this epidemiological context, the implementation of a permanent WNV surveillance system seems necessary. An integrated surveillance system, as the one implemented in the region, can lead to accelerated WNV detection and the prevention of human infections. Moreover, it allows the collection of data that are useful to better understand the epidemiology of WNV infection, and to identify possible risk factors related to human infections.

Joint WNV surveillance efforts of the public and the animal health sectors are resource intensive activities. However, our results can bring evidence on the economic return of such cooperation for zoonoses mitigation in Europe. These results can contribute to build an evidence base for One Health operations, with the aim to guide disease mitigation policy, decision making, and capacity building within a One Health context. Further evaluations including intangible costs, social, and ecological dimensions, would allow a deeper understanding of the economic context of the disease and its mitigation, allowing to better inform public health decision makers.

## Supporting information

S1 Table West Nile neuroivasive disease cases hospitalization dataset. (XLSX)
